# The menin inhibitor revumenib in *KMT2A*-rearranged or *NPM1*-mutant leukaemia

**DOI:** 10.1038/s41586-023-05812-3

**Published:** 2023-03-15

**Authors:** Ghayas C. Issa, Ibrahim Aldoss, John DiPersio, Branko Cuglievan, Richard Stone, Martha Arellano, Michael J. Thirman, Manish R. Patel, David S. Dickens, Shalini Shenoy, Neerav Shukla, Hagop Kantarjian, Scott A. Armstrong, Florian Perner, Jennifer A. Perry, Galit Rosen, Rebecca G. Bagley, Michael L. Meyers, Peter Ordentlich, Yu Gu, Vinit Kumar, Steven Smith, Gerard M. McGeehan, Eytan M. Stein

**Affiliations:** 1 Department of Leukemia, The University of Texas MD Anderson Cancer Center, Houston, TX USA; 2 City of Hope, Duarte, CA USA; 3 Washington University School of Medicine in St. Louis, St. Louis, MO USA; 4 Dana-Farber Cancer Institute, Boston, MA USA; 5 Winship Cancer Institute, Emory University School of Medicine, Atlanta, GA USA; 6 University of Chicago, Chicago, IL USA; 7 Florida Cancer Specialists/Sarah Cannon Research Institute, Sarasota, FL USA; 8 University of Iowa, Iowa City, IA USA; 9 Memorial Sloan Kettering Cancer Center, New York, NY USA; 10 Greifswald University Medical Center, Greifswald, Germany; 11 Syndax Pharmaceuticals, Waltham, MA USA

**Keywords:** Acute myeloid leukaemia, Targeted therapies

## Abstract

Targeting critical epigenetic regulators reverses aberrant transcription in cancer, thereby restoring normal tissue function^1–3^. The interaction of menin with lysine methyltransferase 2A (KMT2A), an epigenetic regulator, is a dependence in acute leukaemia caused by either rearrangement of *KMT2A* or mutation of the nucleophosmin 1 gene (*NPM1*)^4–6^. *KMT2A* rearrangements occur in up to 10% of acute leukaemias and have an adverse prognosis, whereas *NPM1* mutations occur in up to 30%, forming the most common genetic alteration in acute myeloid leukaemia^7,8^. Here, we describe the results of the first-in-human phase 1 clinical trial investigating revumenib (SNDX-5613), a potent and selective oral inhibitor of the menin–KMT2A interaction, in patients with relapsed or refractory acute leukaemia (ClinicalTrials.gov, NCT04065399). We show that therapy with revumenib was associated with a low frequency of grade 3 or higher treatment-related adverse events and a 30% rate of complete remission or complete remission with partial haematologic recovery (CR/CRh) in the efficacy analysis population. Asymptomatic prolongation of the QT interval on electrocardiography was identified as the only dose-limiting toxicity. Remissions occurred in leukaemias refractory to multiple previous lines of therapy. We demonstrate clearance of residual disease using sensitive clinical assays and identify hallmarks of differentiation into normal haematopoietic cells, including differentiation syndrome. These data establish menin inhibition as a therapeutic strategy for susceptible acute leukaemia subtypes.

## Abstract (web summary)

Revumenib, a potent and selective oral inhibitor of the menin–KMT2A interaction, is associated with a low frequency of treatment-related adverse events and promising clinical activity in patients with relapsed or refractory acute leukaemia.

## Main

The prognosis of acute leukaemias harbouring rearrangements of the gene lysine methyltransferase 2A (*KMT2A*), previously known as mixed-lineage leukaemia (*MLL*), is poor, with a 5-year overall survival of less than 25% (ref. ^7^). *KMT2A* rearrangements (*KMT2A*r) occur in 80% of infant acute lymphoblastic leukaemia (ALL) and in 5–15% of children and adults with acute leukaemia, whether myeloid, lymphoid or mixed phenotype^9^. Mutated nucleophosmin 1 gene (*NPM1*) is the most common genetic alteration in adult acute myeloid leukaemia (AML), occurring in up to 30% of patients^8^. Currently there are no targeted therapies specifically approved for acute leukaemia with *KMT2A*r or mutated *NPM1*.

**Extended Data Fig. 1:**
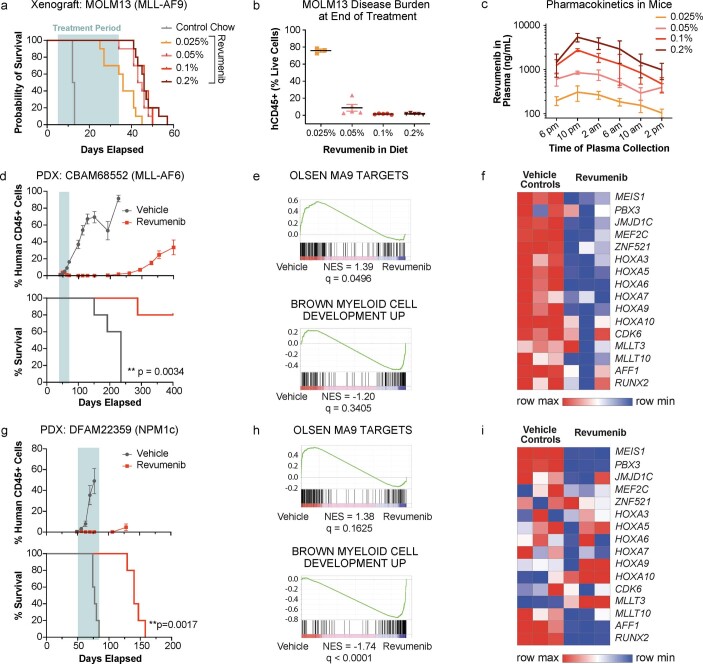
Revumenib suppresses both *KMT2A*r and *NPM1*-mutant AML in preclinical models of leukaemia. NSG mice were engrafted with the *KMT2A*r cell line MOLM-13 (**a**; n = 10) or patient derived xenografts (PDX) harbouring either a *KMT2A*r (**d**; n = 5) or *NPM1* mutation (**g**; n = 5). Mice were treated for 28 days with revumenib, which was formulated in chow, across a dose range of 0.025% to 0.2% (**a**) or at 0.1% fixed dose (**d**, **g**). Leukaemic burden (CD45+) was assessed at end of treatment (**b**; 0.025%: n = 3; 0.05-0.2%: n = 5, data represent mean ± SEM) or throughout the study (**d**, **g;** data represent mean ± SEM). For MOLM-13 engrafted mice, revumenib showed clear dose-dependent exposure (**c**; n = 3 with 3 individual measurements per timepoint and dose, data represent mean ± SD), which translated into dose-responsive effect on survival benefit (**a**) and leukaemic burden at end of treatment (**b**). Similarly, revumenib treatment of the PDX models led to significant suppression of leukaemic burden and significant survival benefit in each (**d**, **g**). *P*-values were determined by log-rank (Mantel–Cox) tests. Adjustments were not made for multiple comparisons. Revumenib treatment also led to broad changes in the transcriptional program (**f**, **i**; n = 3 per treatment group; GEO accession number for RNAseq data, GSE216730) with GSEA results consistent with previously reported signatures (**e**, **h**; n = 3 per treatment group). GSEA, gene set enrichment analysis; NES, normalized enrichment score; NSG, NOD scid gamma; PDX, patient-derived xenograft; SD, standard deviation; SEM, standard error of the mean.

Genetic rearrangements of *KMT2A* lead to aberrant expression of homeobox (*HOX*) genes and their DNA-binding cofactor *MEIS1* (ref. ^10^). This gene expression programme, normally expressed in stem cells, causes a haematopoietic differentiation block and leukaemic transformation^11^. For leukaemias driven by *KMT2A*r, menin is a critical oncogenic cofactor^4^. The menin-binding motif is preserved throughout all KMT2A fusion proteins, and menin is an essential cofactor for binding to *HOX* gene promoters^7^. Similarly, in AML with mutated *NPM1*, the interaction between wild-type *KMT2A* and menin leads to *HOX-* and *MEIS1*-mediated leukaemogenic transcription^6,12^. Blockade of the menin–KMT2A interaction disrupts the assembly of oncogenic KMT2A wild-type or fusion complexes on chromatin^12^. Preclinical studies showed that menin inhibition downregulates *HOX* and *MEIS1* transcription and reverses leukaemogenesis in *KMT2Ar-r-* or *NPM1*-mutated leukaemia models^5,12,13^. Revumenib, previously known as SNDX-5613, is a potent, oral, selective inhibitor of the menin–KMT2A interaction. Treatment with revumenib led to abrogation of aberrant *HOX* gene expression and dramatic antileukaemic activity in those models (Extended Data Fig. 1; GEO accession no. for RNA sequencing (RNA-seq) data, GSE216730). In this first-in-human clinical trial we assessed the safety, maximum tolerated dose (MTD), recommended phase 2 dose (RP2D) and pharmacokinetic and pharmacodynamic profiles in patients with relapsed or refractory acute leukaemia, and present the clinical activity of revumenib in patients with *KMT2A*r or mutated *NPM1*.

**Extended Data Fig. 2:**
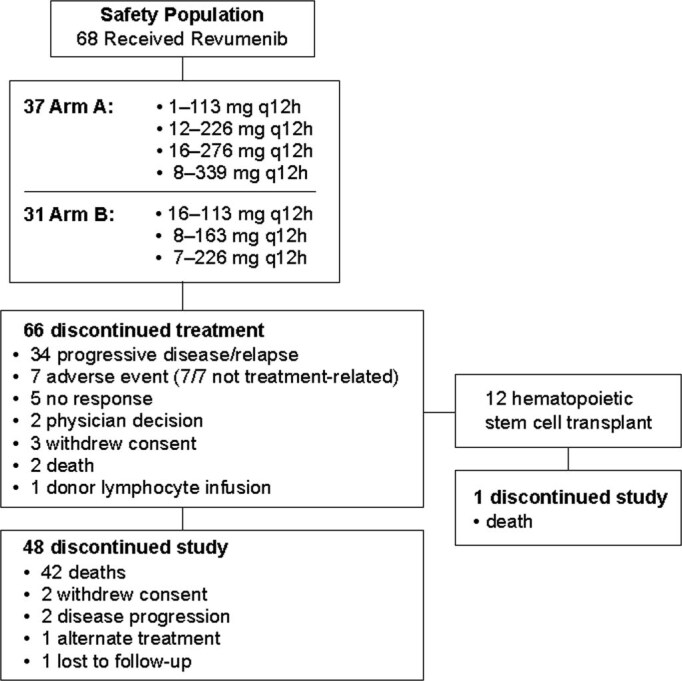
CONSORT diagram and patient disposition on trial. The CONSORT diagram shows the number of patients from Arms A and B who discontinued treatment and lists the reasons for treatment and study discontinuation.

This phase 1 dose-escalation study was conducted across nine sites in the United States. Because revumenib is a substrate of cytochrome P450 3A4 (CYP3A4), two parallel dose-escalation cohorts, one without (Arm A) and one with (Arm B) strong CYP3A4 inhibitors, were conducted. Revumenib was administered orally every 12 h (q12h) in continuous 28-day cycles. Although all patients could enrol at study initiation regardless of cytogenetic and mutational profile, given the strong preclinical rationale demonstrating that the menin–KMT2A interaction is a targetable vulnerability in acute leukaemia with *KMT2A*r or mutated *NPM1*, an early amendment to the protocol restricted enrolment to patients with relapsed or refractory acute leukaemia with *KMT2A*r or mutated *NPM1* (88% of the total study population). Between 5 November 2019 and 31 March 2022, a total of 68 patients (37 in Arm A and 31 in Arm B) were enroled and treated. The data cutoff date for this analysis was 31 March 2022. Patient disposition is presented in Extended Data Fig. 2.

Baseline characteristics of patients are provided in Extended Data Table 1. There were 56 patients (82%) with relapsed or refractory AML, 11 (16%) with ALL and one with mixed-phenotype acute leukaemia (2%). Forty-six patients (68%) had *KMT2A*r, 14 (21%) had mutated *NPM1* and eight (12%) had neither *KMT2A*r nor *NPM1* mutations. Sixty patients were adults (at least 18 years old) and eight were children or adolescents (under 18 years of age). The median age was 42.5 years (range, 0.8–79). The median age of adult patients was 50.5 years (range, 19–79) and the median age among paediatric patients was 2.5 years (range, 0.8–16). The study population was heavily pretreated with a median of four previous lines of therapy (range, 1–12), and 31 patients (46%) had relapsed after an allogenic stem cell transplant. All patients who received at least one dose of revumenib were included in the safety analysis whereas only patients with *KMT2A*r or mutated *NPM1* were included in the efficacy analysis.

**Extended Data Fig. 3:**
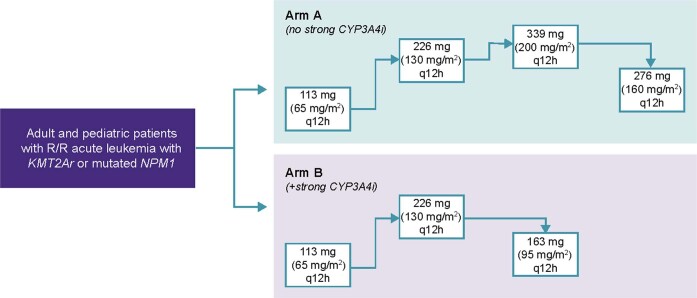
Dose escalation schema. The revumenib dose was adjusted based on the body surface area (BSA) for patients weighing less than 40 kg as indicated for each corresponding dose level shown in parentheses. CYP3A4i, cytochrome P450 3A4 inhibitor; q12h, every 12 h; R/R, relapsed or refractory.

The dose-escalation schema is shown in Extended Data Fig. 3. The only dose-limiting toxicity observed in both Arm A and Arm B was grade 3 prolongation of the QT interval (over 500 ms) on electrocardiography (ECG), a toxicity predicted in preclinical animal studies. These events occurred without any clinical correlate or symptoms. This dose-limiting toxicity was observed in Arm A at dose levels of 226 mg q12h (*n* = 1) and 339 mg q12h (*n* = 2), and in Arm B at 113 mg q12h (*n* = 1) and 226 mg q12h (*n* = 2). The incidence of grade 3 QT prolongation was 10% at dose levels either at or below MTD.

A total of 67 treated patients (99%) had an adverse event during treatment with revumenib whereas 53 patients (78%) had a treatment-related adverse event (TRAE) of any grade (Table 1). The most frequent treatment-emergent adverse events (TEAEs) of any grade, irrespective of a relationship to revumenib, were prolongation of the QT interval (56%), nausea (50%), vomiting (40%) and febrile neutropenia (31%); a full listing of these adverse events in at least 20% of the patients is given in Table 1. The most frequent TEAEs of grade 3 or higher were febrile neutropenia (31%), thrombocytopenia (19%) and sepsis (18%) (Extended Data Table 2). The most frequent TRAE was prolongation of the QT interval, in 36 patients (53%). Eleven patients (16%) had grade 3 or 4 TRAEs (Extended Data Table 2). The most frequent grade 3 TRAE was prolongation of the QT interval, in nine patients (13%). There were no ventricular arrythmias, and all episodes of grade 3 QT prolongation resolved either before the next dose or with protocol-directed dose hold. All patients with grade 3 QT prolongation were able to resume dosing at the next-lower dose level. Other grade 3 TRAEs included diarrhoea (3%), hypercalcaemia (2%), tumour lysis syndrome (2%), anaemia (3%), asthenia (2%), fatigue (2%), neutropenia (2%), thrombocytopenia (2%), and hypokalaemia (2%). Grade 4 TRAEs included neutropenia (2%) and thrombocytopenia (2%), with one event each. No patients permanently discontinued revumenib due to TRAEs, and no TRAEs led to death.

**Table 1:** Any-grade treatment-related and TEAEs, regardless of causality

Event	Overall population (*n* = 68)
**Any-grade TRAE (5% or over)**	53 (77.9%)
ECG QT prolonged	36 (52.9%)
Nausea	18 (26.5%)
Differentiation syndrome	11 (16.2%)
Vomiting	11 (16.2%)
Diarrhoea	7 (10.3%)
Decreased appetite	5 (7.4%)
Dysgeusia	5 (7.4%)
**Any-grade TEAE (20% or over)**	67 (98.5%)
ECG QT prolonged	38 (55.9%)
Nausea	34 (50.0%)
Vomiting	27 (39.7%)
Febrile neutropenia	21 (30.9%)
Diarrhoea	20 (29.4%)
Fatigue	18 (26.5%)
ALT increased	17 (25.0%)
Headache	16 (23.5%)
Hyperphosphataemia	16 (23.5%)
Hypokalaemia	15 (22.1%)
Hyponatraemia	15 (22.1%)
Thrombocytopenia	15 (22.1%)
Epistaxis	14 (20.6%)
Peripheral oedema	14 (20.6%)

All AEs shown as *n* (%).

ALT, alanine aminotransferase.

**Extended Data Fig. 4:**
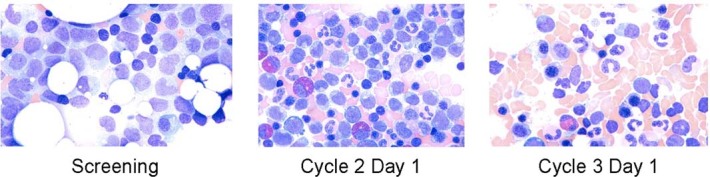
Morphologic evidence of myeloid differentiation. Photomicrographs of bone marrow biopsies demonstrating morphologic evidence of myeloid differentiation in a patient who achieved complete remission; magnification is 40x.

**Extended Data Fig. 5:**
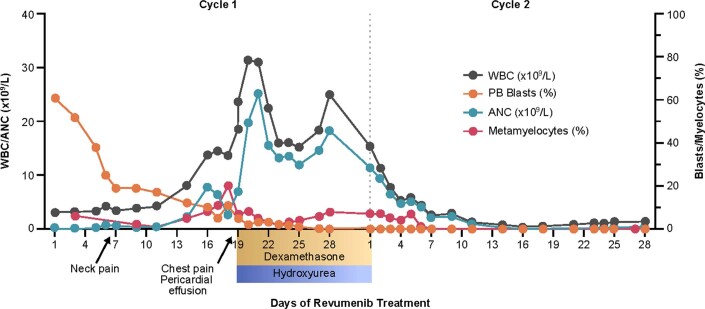
Changes in peripheral blood during differentiation syndrome are associated with the menin inhibitor revumenib. Example from a 71-year-old patient with *KMT2A*r AML relapsed after an allogeneic stem cell transplant, who received revumenib at 339 mg PO q12h (Arm A), and achieved CRh, MRD negative remission. Differentiation syndrome manifested as chest pain with a small pericardial effusion, and a possible prodrome of neck pain likely related to expansion of cervical nodes, all resolved promptly with initiation of steroids followed by tapering doses. Hydroxyurea was used to control leukocytosis. AML, acute myeloid leukaemia; ANC, absolute neutrophil count; CRh, complete remission with partial haematologic recovery; MRD, minimal or measurable residual disease; PB, peripheral blood; PO, by mouth; q12h, every 12 h; WBC, white blood cell.

A common occurrence in antileukaemia therapies that induce myeloid differentiation is the development of a clinically apparent differentiation syndrome. Differentiation syndrome is caused by cytokine alterations associated with haematopoietic differentiation, with common manifestations including fever, arthralgias, leukocytosis, pleural or pericardial effusions and respiratory or renal failure in severe cases^14,15^. This had been mostly reported in patients with myeloid leukaemias treated with all-trans retinoic acid, arsenic trioxide and isocitrate dehydrogenase inhibitors^14–16^. In this study, differentiation syndrome was reported in 11 patients (16%), all grade 2. All cases of differentiation syndrome resolved following treatment with corticosteroids, with the addition of hydroxyurea for associated leukocytosis seen in five of the 11 patients (Extended Data Figs. 4 and 5). One patient missed one day of dosing, but otherwise treatment with revumenib was uninterrupted because of differentiation syndrome. The median time of onset of differentiation syndrome was 18 days (range, 5–41) with manifestations including bone pain or arthralgia, pericardial effusion, pleural effusion, pulmonary infiltrates, increase in creatinine, rash, oedema and pyrexia.

**Extended Data Fig. 6:**
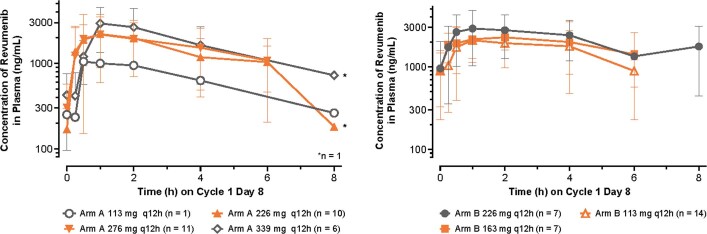
Dose proportional exposure was achieved across both arms. The half-life of revumenib in Arm A (without strong CYP3A4 inhibitors) was approximately 3 h at the cycle 1 day 8 assessment of the 276-mg q12h dose level and was approximately 8 h at the same assessment in Arm B (with a strong CYP3A4 inhibitor) at the 163-mg q12h dose level. Data represent mean ± SD. Data cutoff date for pharmacokinetic analysis was July 11, 2022. CYP3A4, cytochrome P450 3A4; q12h, every 12 h; SD, standard deviation.

Pharmacokinetic studies showed that dose-proportional exposure was achieved in both Arm A (no strong CYP3A4 inhibitor) and Arm B (with a strong CYP3A4 inhibitor). Steady-state levels were achieved in approximately 48 h, with no evidence of drug accumulation (Extended Data Fig. 6).

Following a review of prespecified protocol criteria on safety, tolerability and pharmacokinetic data, the revumenib doses of 226 mg q12h and 276 mg q12h in Arm A and 113 mg q12h and 163 mg q12h in Arm B met the prespecified criteria for RP2D.

**Fig. 1:**
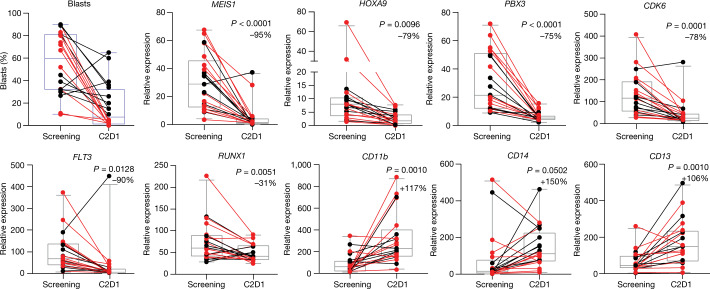
Transcriptional changes following treatment with the menin inhibitor revumenib in patients with relapsed or refractory acute leukaemia with *KMT2A*r or mutated *NPM1*. RNA-seq before and after treatment with revumenib, showing downregulation of critical leukaemogenic target genes *MEIS1*, *HOXA9* and *PBX3* and increase in expression of genes associated with differentiation (*CD11b*, *CD14*), with transcriptional suppression of *FLT3*, a putative transcriptional target of MEIS1. The change in bone marrow blast percentage following treatment is shown. Box plots represent median gene expression or median bone marrow blast percentage, and the 95% CI along with percentage change in gene expression following treatment. Responders are shown in red, nonresponders in black. Results were obtained using a paired *t*-test with a two-sided *P* value. Adjustments were not made for multiple comparisons. This analysis included a cohort of 21 evaluable patients. Revumenib was administered in continious 28-day cycles. C2D1, day 1 of treatment cycle 2.

The pharmacodynamic effects of revumenib were assessed through transcriptional changes following treatment using RNA-seq of bone marrow cells (Fig. 1). Consistent with the established mechanism of action, menin inhibition using revumenib resulted in downregulation of the critical leukaemogenic target genes *MEIS1,* homeobox A9 (*HOXA9*), pre-B-cell leukaemia transcription factor 3 (*PBX3*) and cyclin-dependent kinase 6 (*CDK6*), and an increase in expression of genes associated with differentiation, such as integrin alpha M (*CD11b*) and *CD14*. We also observed transcriptional suppression of fms-like tyrosine kinase 3 (*FLT3*) following treatment, a putative transcriptional target of MEIS1; *FLT3* is frequently mutated in multiple subtypes of AML, including *KMT2A*r AML (10%), and is particularly common in AML with mutated *NPM1* (60%)^8,17–19^.

**Fig. 2:**
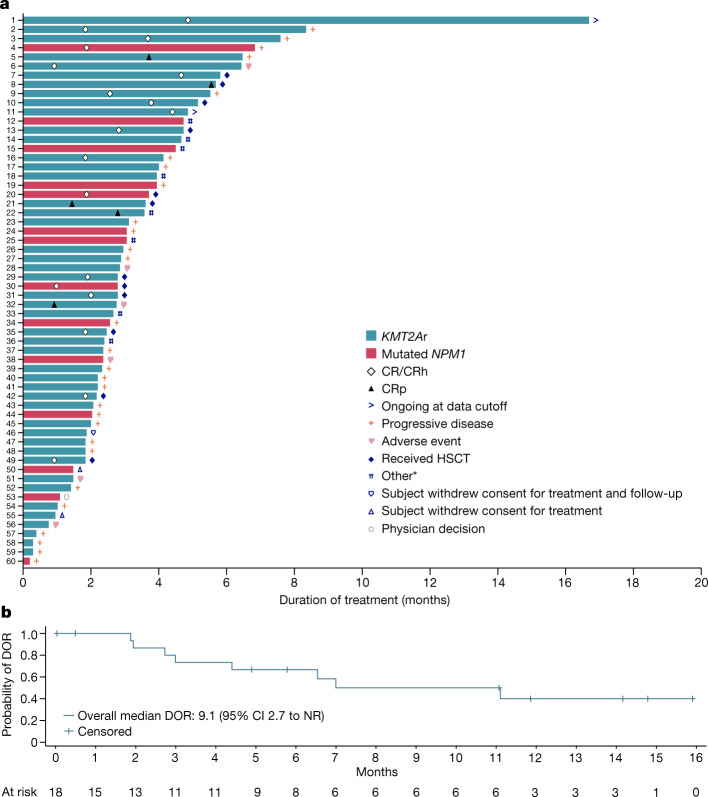
Characterization of remissions with the menin inhibitor revumenib in susceptible relapsed or refractory acute leukaemia subtypes. **a**, Time to response, duration of treatment (censored at time of HSCT) and patient status by the cutoff date. *Other reasons for treatment discontinuation included no response, relapse, death and donor lymphocyte infusion. **b**, Kaplan–Meier curve of duration of response (DOR) in patients with CR or CR/CRh without censoring at the time of an allogeneic stem cell transplant performed in 12 of 18 evaluable patients.

**Extended Data Fig. 7:**
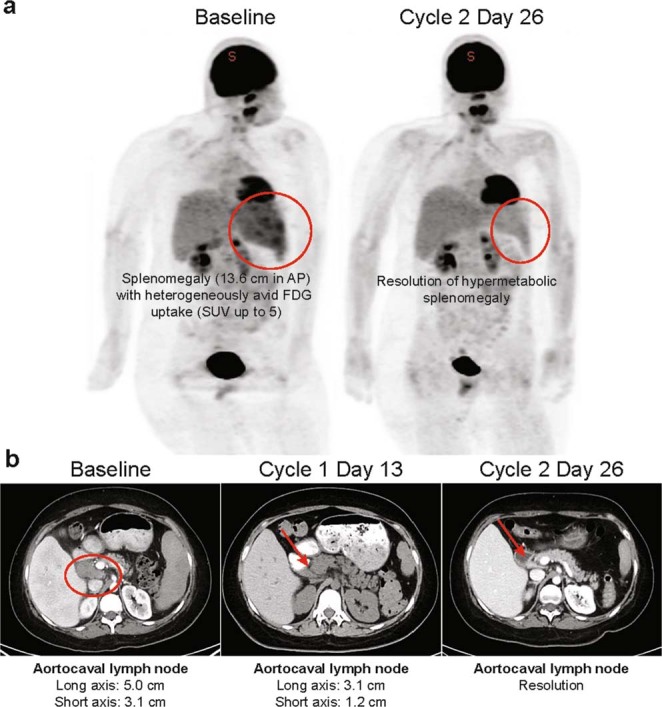
Response in extra-medullary disease. **A**. PET scan at baseline and after 2 cycles of treatment. **B**. Computed tomographic scans of target lesions and spleen from a 19-year-old with relapsed *KMT2A*r AML, previously treated with three prior lines of therapy including 2 allogeneic stem cell transplants and local radiation to the spleen and abdominal nodes, received revumenib at 276 mg PO q12h (Arm A), achieved CRh, MRD negative remission with resolution of extramedullary disease in abdominal nodes and spleen. AML, acute myeloid leukaemia; AP, anterior-posterior; CRh, complete remission with partial haematologic recovery; D, day; FDG, fluorodeoxyglucose; MRD, minimal or measurable residual disease; PET, positron emission tomography; PO, by mouth; q12h, every 12 h; SUV, standardized uptake value.

The rate of complete remission or complete remission with partial haematologic recovery (CR/CRh) was 30% (95% confidence interval (CI) 18.8–43.2) in 18 of 60 evaluable patients with undetectable measurable residual disease (MRD), as assessed by multiparameter flow cytometry, in 14 of 18 patients (78%) who achieved CR/CRh (Fig. 2a and Table 2). The overall response rate (CR/CRh/complete remission with incomplete platelet recovery (CRp)/complete remission with incomplete haematologic recovery (CRi)/morphologic leukaemia-free state) was 53% (32 of 60 evaluable patients). The median time to CR/CRh was 1.9 months (range, 0.9–4.9). Although imaging assessment was not mandated on study, responses were notably seen in both bone marrow and extramedullary sites in two of six evaluable patients with extramedullary leukaemia at enrolment, with representative imaging shown in Extended Data Fig. 7. In an exploratory descriptive analysis, we evaluated responses by both leukaemia lineage and the adult and paediatric (under 18 years of age) populations. Morphologic remissions were identified in 27 of 49 patients (55%) with AML (95% CI 40.2–69.3), in four of ten patients (40%) with ALL (95% CI 12.2–73.8) and in the one patient with a mixed-phenotype acute leukaemia. Morphologic remission was noted in four of eight paediatric patients (50%; 95% CI 15.7–84.3) and in 28 of 52 adults (54%; 95% CI 39.5–67.8).

**Table 2:** Responses to treatment

Response	Efficacy population (*n* = 60)	*KMT2A*r (*n* = 46)	Mutated *NPM1* (*n* = 14)
Overall response^*^	32 (53%)	27 (59%)	5 (36%)
Median time to first morphologic response (range), months	0.95 (0.9–3.7)	0.95 (0.9–3.7)	0.99 (1.0–1.9)
Best response^*^			
CR/CRh	18 (30%)	15 (33%)	3 (21%)
CR	12 (20%)	9 (20%)	3 (21%)
CRh	6 (10%)	6 (13%)	0
Median time to CR or CRh (range), months	1.9 (0.9–4.9)	2.0 (0.9–4.9)	1.9 (1.0–1.9)
CRi	0	0	0
CRp	5 (8%)	5 (11%)	0
MLFS	9 (15%)	7 (15%)	2 (14%)
Partial remission	0	0	0
No response	19 (32%)	12 (26%)	7 (50)
Progressive disease	7 (12%)	6 (13%)	1 (7%)
Missing	2 (3%)	1 (2%)	1 (7%)
MRD^†^ neg. rate within CR/CRh	14/18 (78%)	11/15 (73%)	3/3 (100%)
Median time to MRD^†^ neg. among patients with CR/CRh (range), months	1.9 (0.9–4.9)	1.9 (0.9–4.9)	1.9 (1.0–2.8)

*Responses were assessed by the investigators; responses and MRD-negative rates are shown as *n* (%).

^†^MRD, minimal or measurable residual disease assessed at participating sites by either multicolour flow cytometry or PCR; MRD status percentage based on patients with non-missing MRD status out of all responders.

MLFS, morphologic leukaemia-free state.

In patients with *KMT2A*r who achieved morphologic remission following the first cycle of therapy with revumenib, 21 of 25 (84%) retained the detectable fusions causing *KMT2A*r during concomitant cytogenetic analyses. However, most patients later had clearance of these *KMT2A* rearrangements with subsequent cycles of therapy. The rate of complete cytogenetic response in patients with *KMT2A*r who achieved morphologic clearance of their myeloblasts was 16 of 25 (64%) (Extended Data Table 3); these typically occurred after morphologic remission and, in some cases, after MRD negativity as assessed by immunophenotyping via flow cytometry. The median time to achieving complete cytogenetic response was 1.9 months (range, 0.9–2.8). These findings are consistent with the mechanism of action of differentiation agents, for which response leads first to a phenotypic differentiation of cells such that they retain the genetic fusion but are without aberrant morphology or leukaemia surface marker expression (as detected by flow cytometry)^20,21^. Subsequently with additional cycles following phenotypic remission, fusions are no longer detected by fluorescence in situ hybridization, probably because of apoptosis of differentiated cells or replacement by normal haematopoiesis.

**Extended Data Fig. 8:**
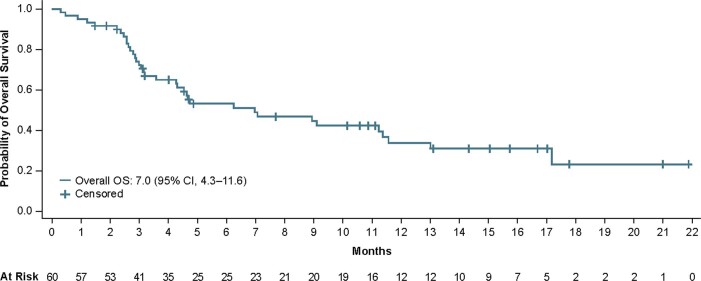
Overall survival in patients with *KMT2A*r or mutated *NPM1*. OS, overall survival.

**Extended Data Fig. 9:**
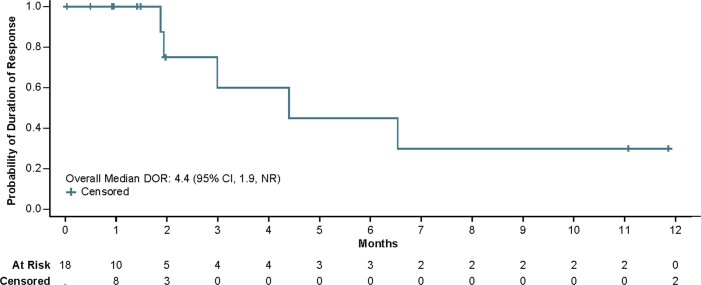
Kaplan-Meier curve of duration of response in patients with complete remission or complete remission with partial haematologic recovery with censoring at time of the allogeneic stem cell transplant. DOR, duration of response; NR, not reached.

With a median follow-up of 11.9 months (95% CI 4.9–14.8) in patients who achieved CR/CRh, the median duration of response was 9.1 months (95% CI 2.7 to not reached (NR)) (Fig. 2b). Median overall survival in the efficacy population, regardless of remission status, was 7 months (95% CI 4.3–11.6) (Extended Data Fig. 8) and median follow-up of overall survival in the efficacy population was 14.3 months (95% CI 10.6–16.7). Twelve patients received an allogeneic stem cell transplant as consolidation following response to revumenib, with nine in remission at the time of the data cutoff, seven of whom have been in remission for over 6 months. The median duration of response in those who achieved CR/CRh with censoring at the time of allogeneic stem cell transplant was 4.4 months (95% CI 1.9–NR) (Extended Data Fig. 9).

**Extended Data Fig. 10:**
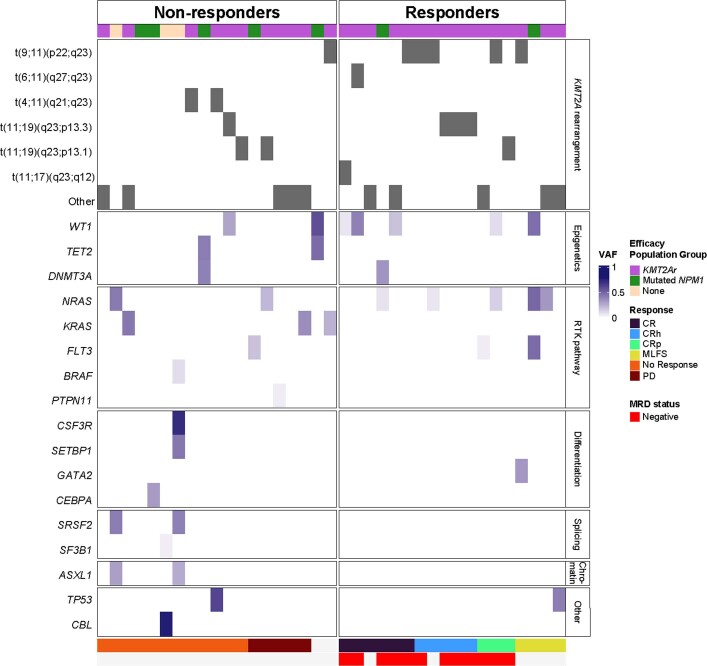
Mutation analysis and response. This cohort included 37 patients evaluable for this analysis. CR, complete remission; CRh, complete remission with partial haematologic recovery; CRp, complete remission with incomplete platelet recovery; MLFS, morphologic leukaemia-free state; NR, not reached; PD, progressive dVAF, variant allele frequency.

Consistent with the preclinical hypothesis regarding the efficacy of menin inhibition in patients with *NPM1* mutations or *KMT2A*r, there were no responses in the eight patients enroled on study who lacked either a *KMT2A*r or *NPM1* mutation. In evaluable patients for whom a targeted sequencing panel was performed at baseline, no specific *KMT2A* translocation or comutation was clearly associated with response in a limited analysis (Extended Data Fig. 10). Notably among evaluable patients with *KMT2A*r or mutated *NPM1*, 14 had a comutation in *FLT3*, of whom 13 had been treated with a FLT3 inhibitor before enrolment. Among the 11 patients with comutated *NPM1* and *FLT3*, three responded whereas two of the three patients with *KMT2A*r and concomitant *FLT3* mutations achieved a response. All responders had previously received a FLT3 inhibitor.

## Discussion

The treatment of patients who have relapsed or refractory acute leukaemia with *KMT2A*r is challenging. The rate of CR or complete remission with incomplete count recovery in relapsed *KMT2A*r AML following two previous lines of therapy is 9% (ref. ^18^). Despite notable progress in the treatment of childhood acute leukaemia, infant *KMT2A*r acute leukaemias have remained a therapeutic challenge with high rates of resistance to multi-agent chemotherapy^22,23^. Mutations in *NPM1* are the most common alterations in AML, and targeting of vulnerabilities associated with this subset expand the reach of targeted therapy to the largest portion of patients with this disease. The menin inhibitor revumenib has the potential to address these unmet needs. In this first-in-human clinical trial, we provide clinical evidence of the effectiveness of menin inhibition with an oral targeted therapy, which is the first epigenetic therapy that evicts protein complexes from chromatin, leading to remissions in patients with acute leukaemia. We found an encouraging clinical benefit, with deep molecular remissions and minimal toxicities, in a heavily pretreated population of both children and adults with advanced acute leukaemia. However, recent data suggest that clinical resistance to targeted menin inhibition is mediated through the acquisition of mutations in menin that prevent inhibitor binding and can lead to clinical relapse^24^. The emergence of resistance mutations highlights the critical role of the menin–KMT2A interaction in the pathogenesis of *KMT2A*r and *NPM1-*mutant acute leukaemia.

Because revumenib disrupts the effect of epigenetic regulators in leukaemia that are dependent on the menin–KMT2A interaction, this leads to abrogation of aberrant gene expression and removal of the haematopoietic differentiation block^12,13^. In this study, we show evidence of this mechanism of action with differentiation of leukaemia cells, persistence of cytogenetic abnormalities at the time of morphologic response and differentiation syndrome associated with menin inhibition in some patients, which resolved following appropriate therapy.

Other leukaemia genotypes have recently been identified as susceptible to menin inhibition, such as AML with rearrangement of the nucleoporin 98 gene (*NUP98*), a common and adverse genotype among children with relapsed and refractory disease^25^. Another example is the rare occurrence of AML with translocations involving the meningioma-1 gene (MN1)^26^. Therefore, menin inhibitors have the potential to reach a larger subset of acute leukaemia with similar dependency on the menin–KMT2A interaction, which could be identified using precision approaches testing characteristic gene expression.

In conclusion, in children and adults with highly refractory acute leukaemia with *KMT2A*r or *NPM1* mutation, menin inhibition with revumenib monotherapy was associated with promising antileukaemic activity leading to deep and sustained remission.

## Methods

### Study design and oversights

We conducted a phase 1, multicentre, open-label, dose-escalation study (ClinicalTrials.gov, NCT04065399). Revumenib was administered orally in either capsule or liquid formulation, q12h, in 28-day continuous cycles with dose adjustment by body surface area for patients weighing under 40 kg (Extended Data Fig. 3). We employed a rolling-6 design, an algorithm-based extension of the 3+3 design that allows for concurrent accrual of two to six patients onto a dose level, thereby shortening the duration of phase 1 without increasing the risk of toxicity^27^. Therefore, the number of patients enrolled followed this dose-escalation design without an absolute prior estimation of sample size. The study included two parallel dose-escalation cohorts, for patients either not taking (Arm A) or taking (Arm B) strong CYP3A4 inhibitors. Dose expansion occurred at safe and efficacious doses. Dose-limiting toxic effects were defined as nonhaematologic toxic effects of grade 3 or higher during cycle 1, or as haematologic toxicities directly attributed to the study drug rather than to the underlying disease.

The study was conducted in accordance with the principles of the Declaration of Helsinki and the International Council for Harmonization Good Clinical Practice. The protocol and amendments were approved by the institutional review board or ethics committee at The University of Texas MD Anderson Cancer Center, City of Hope, Washington University School of Medicine in St. Louis, Dana-Farber Cancer Institute, Winship Cancer Institute, Emory University School of Medicine, University of Chicago, Florida Cancer Specialists/Sarah Cannon Research Institute, University of Iowa and Memorial Sloan Kettering Cancer Center. All patients provided written informed consent. This study was designed by the sponsor (Syndax Pharmaceuticals) in collaboration with the investigators. The data were collected by the investigators and their research staff and were analysed by the sponsor and authors. Drafts of the manuscript were written by the first and last authors and revised in collaboration with all authors and the sponsor, all of whom vouch for the completeness and accuracy of the data and analyses, and for the adherence of the study to the protocol. Assistance in manuscript preparation for submission was provided by a professional service and paid for by the sponsor.

### Patients

An early amendment to the protocol allowed patients aged 30 days and older to be enroled on this study; additionally, it restricted the eligibility criteria from any relapsed or refractory acute leukaemia to only those with *KMT2A*r or mutated *NPM1* in Arms A and B of the study. Mutational status was assessed at each site.

### Study assessments

The primary objectives of the phase 1 portion of this study were to assess safety, the MTD or, if different, RP2D and to characterize the pharmacokinetic parameters of revumenib in arms based on concomitant CYP3A inhibitors. Exploratory objectives included assessment of antileukaemic activity in the efficacy population, which consisted of patients with *KMT2A*r or mutated *NPM1*. All study assessments and analyses included both paediatric and adult patients.

Adverse events were graded with use of the Common Terminology Criteria for Adverse Events, v.5.0. Clinical efficacy was assessed by the investigators with use of a modified version of the 2017 European LeukemiaNet response criteria, to additionally include CRh^28^. Guidelines for managing prolongation of the QT interval included electrolyte repletion and adjustment of revumenib dose. Investigators were encouraged to administer corticosteroids following suspicion of differentiation syndrome based on guidelines included in the protocol. Measurable residual disease assessment was performed in participating institutions using multicolour flow cytometry or PCR^29–31^. In an exploratory mutational analysis we used the ArcherDx VariantPlex Myeloid Panel, a 75-gene, next-generation targeted sequencing product examining genes frequently mutated in myeloid and lymphoid malignancies. Powered by Anchored Multiple PCR chemistry, the panel enables deep strand-specific amplification of unique molecular barcoded DNA fragments for sequencing. Archer Analysis software was used for the analysis and interpretation of sequencing data in the detection of single-nucleotide variants and indels. Variant calling was performed using Invitae’s Comprehensive Targeted Mutation File (v.1.6), and the Vision variant caller to report well-classified variants.

### Statistical analysis

All patients who received at least one dose of revumenib were included in the safety analysis, whereas only those with *KMT2A*r or mutated *NPM1* were included in the efficacy analysis. Time-to-event end points were estimated using the Kaplan–Meier method. Descriptive statistics were used for other clinical, laboratory and pharmacokinetic variables. Clinical data were captured in Medidata Classic Rave 2021.2.0, and data analyses were performed using SAS v.9.4 and GraphPad Prism v.8.0. Changes in gene expression were analysed using a paired *t*-test.

RP2D was determined by the safety review committee based on review of pharmacokinetics, safety and tolerability data among evaluable patients.

### Reporting summary

Further information on research design is available in the Nature Portfolio Reporting Summary linked to this article.

## Online content

Any methods, additional references, Nature Portfolio reporting summaries, source data, extended data, supplementary information, acknowledgements, peer review information; details of author contributions and competing interests; and statements of data and code availability are available at 10.1038/s41586-023-05812-3.

### Supplementary information

Reporting Summary

## Figures and Tables

**Extended Data Table 1:**
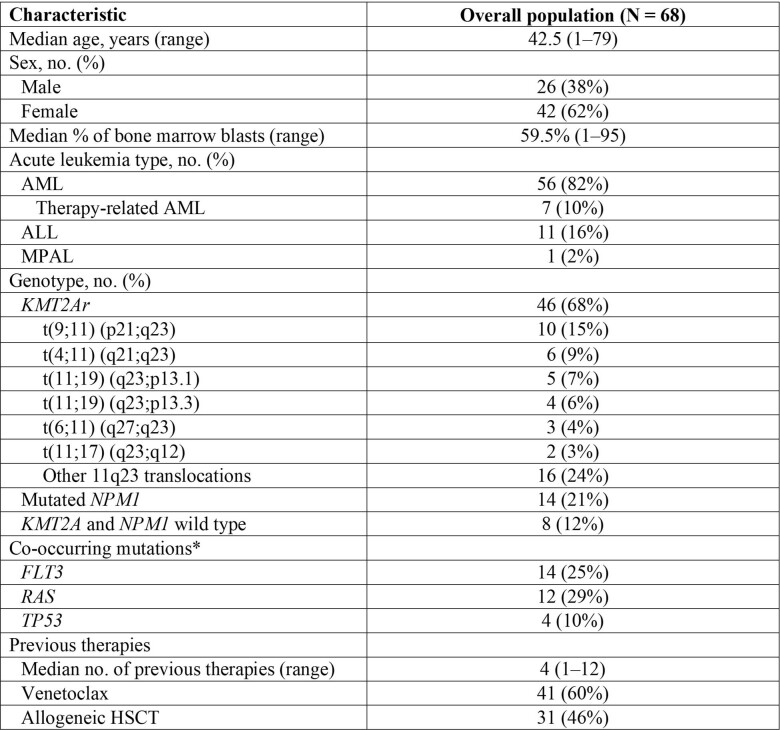
Baseline characteristics

*In patients for whom co-occurring mutation data were available and assessed at each site.

AML, acute myeloid leukaemia; ALL, acute lymphoblastic leukaemia; *FLT3*, fms-related tyrosine kinase*;* HSCT, haematopoietic stem cell transplant; MPAL, mixed phenotype acute leukaemia; *RAS*, rat sarcoma virus.

All values are shown as n (%) except otherwise indicated.

**Extended Data Table 2:**
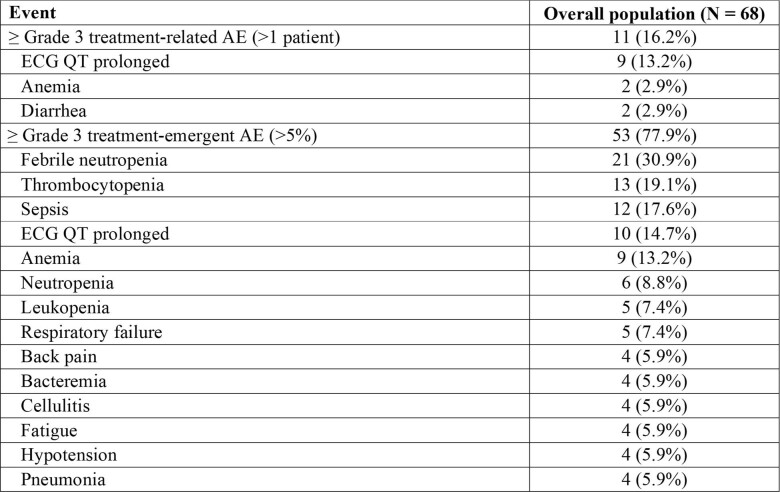
Grade 3 or higher treatment-related and treatment-emergent adverse events

AE, adverse event; ECG, electrocardiogram.

Adverse events are shown as n (%).

**Extended Data Table 3:**
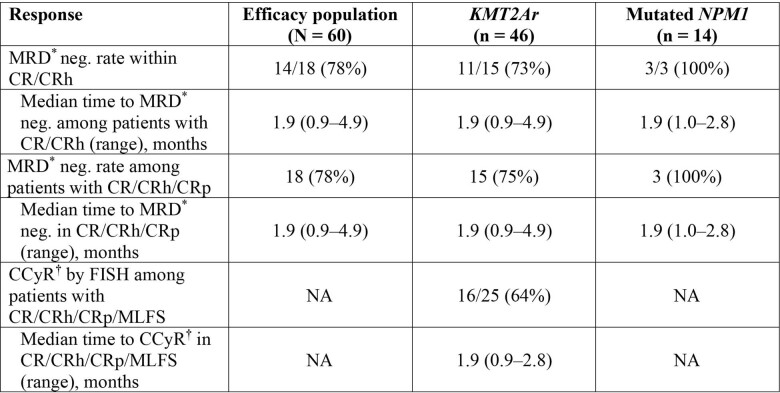
Measurable residual disease and cytogenetic responses

^*^MRD, minimal or measurable residual disease assessed at participating sites by multicolor flow cytometry or by polymerase chain reaction. MRD status percentage is based on patients with non-missing MRD status out of the responders. MRD-negative rates are shown as n (%).

^†^CCyR denotes complete cytogenetic remission in which *KMT2A* fusions were not detectable by fluorescence in situ hybridization in evaluable patients assessed at participating sites.

CR, complete remission; CRh, complete remission with partial haematologic recovery; CRi, complete remission with incomplete haematologic recovery; CRp, complete remission with incomplete platelet recovery; FISH, fluorescence in situ hybridization; MLFS, morphologic leukaemia free state; MRD, minimal or measurable residual disease; NA, not applicable given absence of disease defining cytogenetic abnormalities.

## Data Availability

Syndax Pharmaceuticals (Syndax) is committed to providing qualified scientific researchers access to anonymized data and clinical study reports from the company’s clinical trials for the purpose of conducting legitimate scientific research. Syndax is also obligated to protect the rights and privacy of trial participants and, as such, has a procedure in place for evaluating and fulfilling requests for sharing company clinical trial data with qualified external scientific researchers. Following submission of a request (to datarequest@syndax.com), Syndax will provide an outline of the process and requirements for submitting a data request. Feasible requests will be reviewed by a committee of Syndax subject matter experts to assess the scientific validity of the request and the qualifications of the requestors. In line with data privacy legislation, submitters of approved requests must enter into a standard data-sharing agreement with Syndax before Syndax may grant data access. Data will be made available for request after product approval in the United States and European Union, or after product development is discontinued. There are circumstances that may prevent Syndax from sharing requested data, including country- or region-specific regulations. If Syndax declines the request, it will communicate the decision to the investigator. Access to genetic or exploratory biomarker data requires a detailed statistical analysis plan that is collaboratively developed by the requester and Syndax subject matter experts.
