# Supplementary material for: The menin inhibitor revumenib in KMT2A-rearranged or NPM1-mutant leukaemia
Source: Nature. 2023 Mar 15;615(7954):920–4. doi: 10.1038/s41586-023-05812-3 (PMC10060155; doi:10.1038/s41586-023-05812-3)
Supplement: Supplementary file 1 — Reporting Summary [file 41586_2023_5812_MOESM1_ESM.pdf]

Corresponding author(s): Ghayas C. Issa, Eytan M. Stein

Last updated by author(s): Nov 30, 2022

Reporting Summary

Nature Portfolio wishes to improve the reproducibility of the work that we publish. This form provides structure for consistency and transparency in reporting. For further information on Nature Portfolio policies, see our Editorial Policies and the Editorial Policy Checklist.

Statistics

For all statistical analyses, confirm that the following items are present in the figure legend, table legend, main text, or Methods section.

n/a	Confirmed	
☐	☒	The exact sample size (*n*) for each experimental group/condition, given as a discrete number and unit of measurement
☒	☐	A statement on whether measurements were taken from distinct samples or whether the same sample was measured repeatedly
☐	☒	The statistical test(s) used AND whether they are one- or two-sided<br>*Only common tests should be described solely by name; describe more complex techniques in the Methods section.*
☐	☒	A description of all covariates tested
☐	☒	A description of any assumptions or corrections, such as tests of normality and adjustment for multiple comparisons
☐	☒	A full description of the statistical parameters including central tendency (e.g. means) or other basic estimates (e.g. regression coefficient) AND variation (e.g. standard deviation) or associated estimates of uncertainty (e.g. confidence intervals)
☐	☒	For null hypothesis testing, the test statistic (e.g. *F*, *t*, *r*) with confidence intervals, effect sizes, degrees of freedom and *P* value noted<br>*Give P values as exact values whenever suitable.*
☒	☐	For Bayesian analysis, information on the choice of priors and Markov chain Monte Carlo settings
☒	☐	For hierarchical and complex designs, identification of the appropriate level for tests and full reporting of outcomes
☒	☐	Estimates of effect sizes (e.g. Cohen's *d*, Pearson's *r*), indicating how they were calculated

Our web collection on statistics for biologists contains articles on many of the points above.

Software and code

Policy information about availability of computer code

Data collection	The clinical data were captured in Medidata Classic Rave® 2021.2.0
Data analysis	SAS version 9.4 was used to analyze clinical data, and GraphPad Prism version 8.0. Changes in gene expression

For manuscripts utilizing custom algorithms or software that are central to the research but not yet described in published literature, software must be made available to editors and reviewers. We strongly encourage code deposition in a community repository (e.g. GitHub). See the Nature Portfolio guidelines for submitting code & software for further information.

Data

Policy information about availability of data

All manuscripts must include a data availability statement. This statement should provide the following information, where applicable:

- Accession codes, unique identifiers, or web links for publicly available datasets
- A description of any restrictions on data availability
- For clinical datasets or third party data, please ensure that the statement adheres to our policy

Syndax Pharmaceuticals, Inc. (Syndax) is committed to providing qualified scientific researchers access to anonymized data and clinical study reports from the company's clinical trials for the purpose of conducting legitimate scientific research. Syndax is also obligated to protect the rights and privacy of trial participants and, as such, has a procedure in place for evaluating and fulfilling requests for sharing company clinical trial data with qualified external scientific researchers. Upon submission of a request to datarequest@syndax.com, Syndax will provide an outline of the process and requirements for submitting a data request. Feasible

requests will be reviewed by a committee of Syndax subject matter experts to assess the scientific validity of the request and the qualifications of the requestors. In line with data privacy legislation, submitters of approved requests must enter into a standard data-sharing agreement with Syndax before Syndax may grant data access. Data will be made available for request after product approval in the US and EU or after product development is discontinued. There are circumstances that may prevent Syndax from sharing requested data, including country or region-specific regulations. If Syndax declines the request, it will communicate the decision to the investigator. Access to genetic or exploratory biomarker data requires a detailed statistical analysis plan that is collaboratively developed by the requestor and Syndax subject matter experts.

Human research participants

Policy information about studies involving human research participants and Sex and Gender in Research.

Reporting on sex and gender	Sex and gender-based analyses were not performed given the lack of statistical power to infer meaningful conclusions in subgroup analyses of this first-in-human phase 1 study.
Population characteristics	Key eligibility criteria include age 30 days or older; relapsed or refractory acute leukemia with KMT2A rearranged or mutated NPM1 performed at each participating site; have Eastern Cooperative Oncology Group (ECOG) performance status score 0–2 (if aged ≥18 years); Karnofsky Performance Scale of ≥50 (if aged ≥16 years and <18 years); Lansky Performance Score of ≥50 (if aged <16 years); have adequate organ function. Extended data table 1 summarizes the baseline demographics and disease characteristics for the overall population enrolled. More details are available in the protocol.
Recruitment	Patients were recruited in participating centers (City of Hope, Duarte, CA; Florida Cancer Specialists/Sarah Cannon Research Institute, Sarasota, FL; Memorial Sloan Kettering Cancer Center, New York, NY; The University of Texas MD Anderson Cancer Center, Houston, TX; University of Chicago, Chicago, IL; University of Iowa, Iowa City, IA; Washington University School of Medicine in St. Louis, St. Louis, MO; Winship Cancer Institute, Emory University School of Medicine, Atlanta, GA) in the United States according to the full protocol eligibility criteria without bias.
Ethics oversight	The study protocol and all amendments were approved by the institutional review board or ethics committee at The University of Texas MD Anderson Cancer Center, City of Hope, Washington University School of Medicine in St. Louis, Dana-Farber Cancer Institute, Winship Cancer Institute, Emory University School of Medicine, University of Chicago, Florida Cancer Specialists/Sarah Cannon Research Institute, University of Iowa, and Memorial Sloan Kettering Cancer Center.

Note that full information on the approval of the study protocol must also be provided in the manuscript.

Field-specific reporting

Please select the one below that is the best fit for your research. If you are not sure, read the appropriate sections before making your selection.

☒ Life sciences ☐ Behavioural & social sciences ☐ Ecological, evolutionary & environmental sciences

For a reference copy of the document with all sections, see nature.com/documents/nr-reporting-summary-flat.pdf

Life sciences study design

All studies must disclose on these points even when the disclosure is negative.

Sample size	Dose escalation in this phase 1 study employed a Rolling 6 trial design (Skolnik 2008). The study included two parallel dose escalation cohorts, for patients not taking (Arm A) or taking (Arm B) strong CYP3A4 inhibitors, where 2 to 6 patients can be concurrently enrolled into a dose level, dependent upon (1) the number of patients enrolled at the current dose level; (2) the number of patients who have experienced dose-limiting toxicity (DLT) at the current dose level; and (3) the number of patients entered but with tolerability data pending at the current dose level. Accrual is suspended when a cohort of 6 has enrolled or when the study endpoints have been met. A dose cohort in either arms may be expanded to 12 patients.
Data exclusions	No data from the specified efficacy or safety populations were excluded from analysis.
Replication	This is a phase 1 clinical trial which enrolled eligible human subjects with appropriate sample size calculation, therefore replication of data is not applicable for analysis of trial results.
Randomization	This is a phase 1 clinical trial, no randomization of subjects performed between arms (with and without a CYP3A inhibitor) in order to allow patients to receive the anti-fungal that fits best their clinical need.
Blinding	This is a single arm study, blinding is not applicable.

Reporting for specific materials, systems and methods

We require information from authors about some types of materials, experimental systems and methods used in many studies. Here, indicate whether each material, system or method listed is relevant to your study. If you are not sure if a list item applies to your research, read the appropriate section before selecting a response.

Materials & experimental systems

n/a	Involved in the study
☒	☐ Antibodies
☐	☒ Eukaryotic cell lines
☒	☐ Palaeontology and archaeology
☒	☐ Animals and other organisms
☐	☒ Clinical data
☒	☐ Dual use research of concern

Methods

n/a	Involved in the study
☒	☐ ChIP-seq
☒	☐ Flow cytometry
☐	☐ MRI-based neuroimaging

Eukaryotic cell lines

Policy information about cell lines and Sex and Gender in Research

Cell line source(s)	MOLM-13 were acquired from DSMZ (Deutsche Sammlung von Mikroorganismen und Zellkulturen GmbH).
Authentication	The cells were not authenticated.
Mycoplasma contamination	Cells tested negative for mycoplasma contamination.
Commonly misidentified lines (See ICLAC register)	No commonly misidentified cell lines were used in this study.

Clinical data

Policy information about clinical studies

All manuscripts should comply with the ICMJE guidelines for publication of clinical research and a completed CONSORT checklist must be included with all submissions.

Clinical trial registration	ClinicalTrials.gov, NCT04065399
Study protocol	Available in the supplementary information section.
Data collection	Data were collected at participating centers in the United States between November 5, 2019, and March 31, 2022 (City of Hope, Duarte, CA; Florida Cancer Specialists/Sarah Cannon Research Institute, Sarasota, FL; Memorial Sloan Kettering Cancer Center, New York, NY; The University of Texas MD Anderson Cancer Center, Houston, TX; University of Chicago, Chicago, IL; University of Iowa, Iowa City, IA; Washington University School of Medicine in St. Louis, St. Louis, MO; Winship Cancer Institute, Emory University School of Medicine, Atlanta, GA). The data cutoff date for this analysis was March 31, 2022.
Outcomes	The primary endpoints of the this phase 1 trial are: (1) To determine the safety, tolerability, the maximum tolerated dose (MTD), or, if different, the recommended phase 2 dose (RP2D) in arms A (without strong CYP3A4 inhibitors), and B (with strong CYP3A4 inhibitors). (2) To characterize the pharmacokinetic parameters of SNDX-5613. Exploratory objectives included assessment of the antileukemic activity of SNDX-5613 and an evaluation of the relationship between clinical or pharmacodynamic biomarkers with safety and efficacy. Dose-limiting toxic effects were defined as non-hematologic toxic effects of grade 3 or higher during cycle 1 or hematologic toxicities directly attributed to study drug and not to the underlying disease. Adverse events were graded with the use of the Common Terminology Criteria for Adverse Events (CTCAE), version 5.0. Clinical efficacy was assessed by the investigators with the use of a modified version of the 2017 European LeukemiaNet response criteria including complete remission with partial hematologic recovery (CRh). More details are available in the protocol.

Magnetic resonance imaging

Experimental design

Design type	Not applicable
Design specifications	Not applicable
Behavioral performance measures	Not applicable

Acquisition

Imaging type(s) — *Specify: functional, structural, diffusion, perfusion.*

Field strength — *Specify in Tesla*

Sequence & imaging parameters — *Specify the pulse sequence type (gradient echo, spin echo, etc.), imaging type (EPI, spiral, etc.), field of view, matrix size, slice thickness, orientation and TE/TR/flip angle.*

Area of acquisition — *State whether a whole brain scan was used OR define the area of acquisition, describing how the region was determined.*

Diffusion MRI ☐ Used ☐ Not used

Preprocessing

Preprocessing software — *Provide detail on software version and revision number and on specific parameters (model/functions, brain extraction, segmentation, smoothing kernel size, etc.).*

Normalization — *If data were normalized/standardized, describe the approach(es): specify linear or non-linear and define image types used for transformation OR indicate that data were not normalized and explain rationale for lack of normalization.*

Normalization template — *Describe the template used for normalization/transformation, specifying subject space or group standardized space (e.g. original Talairach, MNI305, ICBM152) OR indicate that the data were not normalized.*

Noise and artifact removal — *Describe your procedure(s) for artifact and structured noise removal, specifying motion parameters, tissue signals and physiological signals (heart rate, respiration).*

Volume censoring — *Define your software and/or method and criteria for volume censoring, and state the extent of such censoring.*

Statistical modeling & inference

Model type and settings — *Specify type (mass univariate, multivariate, RSA, predictive, etc.) and describe essential details of the model at the first and second levels (e.g. fixed, random or mixed effects; drift or auto-correlation).*

Effect(s) tested — *Define precise effect in terms of the task or stimulus conditions instead of psychological concepts and indicate whether ANOVA or factorial designs were used.*

Specify type of analysis: ☐ Whole brain ☐ ROI-based ☐ Both

Statistic type for inference (See Eklund et al. 2016) — *Specify voxel-wise or cluster-wise and report all relevant parameters for cluster-wise methods.*

Correction — *Describe the type of correction and how it is obtained for multiple comparisons (e.g. FWE, FDR, permutation or Monte Carlo).*

Models & analysis

n/a	Involved in the study
☐	☐ Functional and/or effective connectivity
☐	☐ Graph analysis
☐	☐ Multivariate modeling or predictive analysis

Functional and/or effective connectivity — *Report the measures of dependence used and the model details (e.g. Pearson correlation, partial correlation, mutual information).*

Graph analysis — *Report the dependent variable and connectivity measure, specifying weighted graph or binarized graph, subject- or group-level, and the global and/or node summaries used (e.g. clustering coefficient, efficiency, etc.).*

Multivariate modeling and predictive analysis — *Specify independent variables, features extraction and dimension reduction, model, training and evaluation metrics.*
